# Increased Serum Type I Interferon Activity in Organ-Specific Autoimmune Disorders: Clinical, Imaging, and Serological Associations

**DOI:** 10.3389/fimmu.2013.00238

**Published:** 2013-08-19

**Authors:** Clio P. Mavragani, Timothy B. Niewold, Antonis Chatzigeorgiou, Stamatina Danielides, Dimitrios Thomas, Kyriakos A. Kirou, Elli Kamper, Grigorios Kaltsas, Mary K. Crow

**Affiliations:** ^1^Mary Kirkland Center for Lupus Research, Hospital for Special Surgery, New York, NY, USA; ^2^Department of Physiology, School of Medicine, University of Athens, Athens, Greece; ^3^Division of Rheumatology and Department of Immunology, Mayo Clinic, Rochester, MN, USA; ^4^Department of Pathophysiology, School of Medicine, University of Athens, Athens, Greece; ^5^Molecular Physiology and Therapeutics Branch, National Institute of Dental and Craniofacial Research, National Institutes of Health, Bethesda, MD, USA

**Keywords:** type I interferon, autoimmune thyroid disease, organ-specific autoimmunity, type I diabetes

## Abstract

**Background:** Activation of the type I interferon (IFN) pathway has been implicated in the pathogenesis of systemic autoimmune disorders but its role in the pathogenesis of organ-specific autoimmunity is limited. We tested the hypothesis that endogenous expression of type I IFN functional activity contributes to the pathogenesis of autoimmune thyroid disease (ATD) and type I diabetes (T1DM).

**Methods:** We studied 39 patients with ATD and 39 age and sex matched controls along with 88 T1DM patients and 46 healthy matched controls respectively. Available clinical and serological parameters were recorded by chart review, and thyroid ultrasound was performed in 17 ATD patients. Type I IFN serum activity was determined in all subjects using a reporter cell assay. The rs1990760 SNP of the interferon-induced helicase 1 gene was genotyped in ATD patients.

**Results:** Serum type I IFN activity was increased in patients with ATD and T1DM compared to controls (*p*-values: 0.002 and 0.04, respectively). ATD patients with high type I IFN serum activity had increased prevalence of antibodies against thyroglobulin (anti-Tg) and cardiopulmonary manifestations compared to those with low IFN activity. Additionally, the presence of micronodules on thyroid ultrasound was associated with higher type I IFN levels. In patients with T1DM, high IFN levels were associated with increased apolipoprotein-B levels.

**Conclusion:** Serum type I IFN activity is increased in ATD and T1DM and is associated with specific clinical, serological, and imaging features. These findings may implicate type I IFN pathway in the pathogenesis of specific features of organ-specific autoimmunity.

## Introduction

Autoimmune thyroid diseases (ATD), including Hashimoto’s thyroiditis (HT) and Graves’ disease (GD), as well as type I Diabetes Mellitus (T1DM) are prototype organ-specific autoimmune disorders characterized by loss of immunological tolerance against thyroid and β-cell pancreatic antigens, lymphocytic infiltration of the thyroid gland and the insulin producing pancreatic islands, and various degrees of organ dysfunction (1, 2).

Autoimmune thyroid disease and T1DM share common features with systemic autoimmune disorders, such as multifactorial etiology involving both genetic and environmental factors, female predominance (ATD), and familial aggregation associated with other organ-specific or systemic autoimmune disorders (3–5). Despite the fact that ATD is classically considered as a disease that predominantly affects the thyroid gland, non-specific systemic features such as musculoskeletal complaints, sicca symptomatology, pregnancy loss, and various neurological manifestations may also occur (6). Taken together, these observations suggest that clinically different autoimmune phenotypes might share common pathogenetic pathways.

While increasing evidence over the last few years suggests a dominant role for the type I interferon (IFN) pathway in the pathogenesis of many systemic autoimmune disorders such as systemic lupus erythematosus (SLE) and Sjogren’s syndrome (7, 8), limited data are available regarding the role of the IFN-α pathway in the pathogenesis of organ-specific autoimmune disorders (5). Recent studies have suggested that the Ala946Thr polymorphism of the interferon-induced helicase 1 gene (IFIH1) (SNP ID rs1990760) is associated with type I diabetes (T1DM), GD, and Addison’s disease (9, 10). Recent data also support the protective role of rarer IFIH1 alleles against T1DM (10). The IFIH1 gene, also known as the melanoma differentiation-associated 5 (MDA-5), encodes a putative RNA helicase implicated in sensing of viral RNA and generation of antiviral responses (11). In SLE, risk alleles of the IRF5, IRF7, and IFIH1 genes have been associated with high type I IFN levels and distinct autoantibody profiles (12, 13).

Given that development of thyroid autoimmunity and to a lesser extent T1DM, either separately or in combination, has been previously described after IFN-α treatment (14–18), we hypothesized that activation of the type I IFN pathway may contribute to the pathogenesis of these organ-specific autoimmune disorders. To test this hypothesis, type I IFN activity was measured in sera of patients with ATD, T1DM, and healthy controls (HC), using a sensitive functional assay, and its presence was related to various clinical, biochemical, morphological, and genetic indices.

## Patients and Methods

### Study participants

Thirty-nine patients with ATD (13 with GD and 26 with HT) and 39 age and sex matched HC without evidence of underlying autoimmune disease along with 88 patients with T1DM and 46 HC matched for sex and age were studied. Study participants were followed in the Department of Pathophysiology, University of Athens (ATD patients), and the General Pediatric Hospital Ag.Sophia (TIDM patients) (19). Study subjects signed an informed consent form prior to enrollment in the study. All patients underwent a complete medical history and physical examination. Baseline hematological and biochemical profiles were performed and detailed medical therapy was recorded in all patients.

All ATD participants completed a specific questionnaire addressing symptoms/signs related to systemic autoimmune diseases. Symptoms/signs and parameters recorded included skin manifestations, musculoskeletal features, Raynaud’s phenomenon, sicca symptoms, renal involvement, hematological manifestations (autoimmune hemolytic anemia, leucopenia, thrombocytopenia) cardiovascular and/or pulmonary features (pulmonary hypertension, pulmonary fibrosis, pleuritis, pericarditis, coronary artery disease), and neurological complications (headaches, stroke, white matter microangiopathy, transverse myelitis, cranial/peripheral neuropathy). The presence or absence of autoantibodies to thyroid antigens, including antibodies to thyroglobulin (anti-Tg) and thyroid peroxidase (anti-TPO), thyroid stimulating hormone receptor (TSHR), as well as thyroid stimulating hormone (TSH) levels at the time of diagnosis were also recorded. The normal range of TSH values was 0.5–5 (mU/L). On this basis, TSH levels were defined as high and low (>5 and<0.5 mU/L, respectively).

Patients with T1DM were suffering from no other disease and/or DM related complications and were not taking any medications other than insulin. Biochemical parameters that were particularly recorded in patients with T1DM included cholesterol, triglycerides, HbA1c, apolipoprotein-A and -B as well C-reactive protein (CRP) levels.

Serum from ATD and T1DM patients and controls was collected and stored at −80°C until assayed. Informed consent was obtained from ATD patients and controls as well as from the parents of both diabetic and healthy subjects, according to the Declaration of Helsinki. The study has been approved by the Ethics Committee of Athens University Medical School.

### Serum type I IFN activity

Type I IFN activity was measured in sera derived from ATD, T1DM, and HC using a reporter cell assay, which measures the ability of serum to upregulate IFN-inducible genes in an IFN sensitive cell line as previously described (20). In brief, cells of the WISH epithelial cell line (ATCC) express the type I IFN receptor and are highly responsive to type I IFN. WISH cells were plated at a density of cells/mL in 96 well plates in Minimal Essential Media (Cellgro, Herndon, VA, USA) with 10% fetal calf serum (FCS). The cells were then cultured with 50% patient serum for 6 h. Recombinant human IFN-α (IFNaA; BioSource International, Camarillo, CA, USA) and media were used as positive and negative controls respectively. Subsequently, total cellular mRNA was purified from stimulated cells at the end of the culture period using the Qiagen TurboCapture oligo-dT coated 96 well plate system as per manufacturer protocol (Qiagen, Valencia, CA, USA) and was reverse-transcribed to cDNA immediately following purification using the Superscript III reverse transcriptase system from Invitrogen (Carlsbad, CA, USA). Quantitative real-time polymerase chain reaction (PCR) was then used to quantify specific cDNAs using the Bio-Rad SYBR Green intercalating fluorophore system with a Bio-Rad I-cycler thermocycler and fluorescence detector (Bio-Rad, Hercules, CA, USA). Primers for genes highly induced by type I IFN signaling-interferon induced with tetratricopeptide repeats 1 (IFIT-1, Forward CTCCTTGGGTTCGTCTATAAATTG; Reverse AGTCAGCAGCCAGTCTCAG), Protein kinase R (PKR) (Forward CTTCCATCTGACTCAGGTTT; Reverse TGCTTCTGACGGTATGTATTA), interferon-induced protein with tetratricopeptide repeats 3 (IFIT-3, Forward GGCAGACAGGAAGACTTCTGAAGAACA; Reverse TGACTGCCCTCT-GTGTCTCTGCT), myxovirus (influenza virus) resistance 1 (MX-1, Forward TACCAGGACTACGAGATTG-Reverse TGCCAGGAAGGTCTATTAG) were used in the PCR reaction on the WISH cell derived cDNAs. The housekeeping gene Glyceraldehyde-3-phosphate dehydrogenase (GAPDH, Forward CAACGGATTTGGTCGTATT; Reverse GATGGCAACAA-TATCCACTT) was also quantified in the cDNA samples to control for background gene expression. The type I IFN-induced genes are compared with housekeeping gene expression to determine relative expression. The relative expression is then normalized to the relative expression of the respective genes in unstimulated cells from the same population. Type I IFN activity was calculated as the average relative expression of IFN-inducible genes (IFIT-1 and PKR in the thyroid cohort and IFIT-3, PKR, MX-1 in the diabetes cohort). The cut-off for high serum type I IFN activity among patient samples was defined as the mean plus 1 SD of the IFN activity score of sera from healthy donors (HD) (cut-off for high activity = 1.29, for ATD patients and 0.87 for T1DM patients).

### IFIH1 genotyping

The rs1990760 SNP in IFIH1 was genotyped in ATD patients using real-time PCR with Applied Biosystems Assays-by-Design Taqman primer and allele specific fluorescent labeled probes. Reactions were run using 10 ng of genomic DNA along with primers and probes on an ABI 7900HT PCR machine per manufacturer protocol. Genotype calls were made from clustering diagrams at>99% certainty, and the call rate was>90%.

### Autoantibody assays

Anti-Ro/SSA and anti-RNP/Sm antibodies in the ATD patients were determined by commercial ELISA (Diamedix, FL, USA). Anti-Tg and anti-TPO autoantibodies were measured in the same laboratory using a two-site immunoluminometric assay (DiaSorin, LIASON analyzer, normal range:<100 and<25 IU/mL respectively). TRAbs were measured using commercial kit (DiaSorin Inc., Stillwater, MN, USA, cut-off value: 10%).

### Thyroid ultrasonography

Thyroid ultrasonography was carried out in 17 ATD patients using a high-resolution apparatus (Logic-Book XP, General Electric Co., USA) equipped with a 6–11 MHz broadband linear array probe by a single operator who was unaware of the diagnosis.

High-sensitivity color flow Doppler sonography was used to estimate the intraparenchymal blood flow pattern. The vascularity index and the hypoechogenicity index were calculated for all images as previously described (21). Thyroid volume was measured and the presence of nodules was also recorded [micronodules (diameter <10 mm) and macronodules (diameter >10 mm)]. In addition, thyroid blood flow was also measured at the inferior thyroid artery (16).

### Statistical analysis

Two-group comparisons of continuous data were assessed using unpaired *t*-test on non-parametric Mann–Whitney test for normally or not normally distributed data respectively. Fisher’s exact two-tailed test was used for categorical variables. Correlations between quantitative variables were performed by Spearman’s rho test. Our multivariate analysis consisted of a stepwise logistic regression that was used to identify independent variables that could be associated with high or low type I IFN serum activity in ATD patients. The variables entered in the multivariate model were those shown to be statistically significantly different between high and low IFN groups based on the bivariate analysis (*p* < 0.05).

## Results

### ATD cohort

#### Type I IFN activity in ATD patients and controls

**Figure 1:**
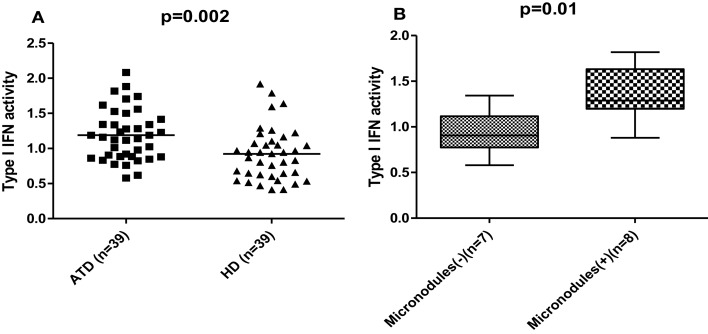
**(A)** Increased type I interferon (IFN) activity in patients with autoimmune thyroid disease (ATD) compared to healthy donors (HD). Serum type I IFN activity was assessed using a sensitive reporter cell assay in 39 patients with autoimmune thyroid disease (ATD) and 39 age-sex matched healthy controls (HC). Results are expressed as an IFN score, as described in materials and methods. Symbols represent individual subjects; horizontal lines represent the mean; *p*-values were calculated by unpaired *t*-test. **(B)** Significantly higher serum type I IFN activity levels in ATD patients with ultrasonographic presence of thyroid micronodules. Serum type I IFN activity assessed using a sensitive reporter cell assay was found to be higher in ATD patients characterized by the presence of micronodules on ultrasound (*n* = 8) compared to those without such nodules (*n* = 7). Data are shown as box plots. Each box represents the 25–75th percentiles. Lines inside the box represent the median. Lines outside the box represent the 10th and the 90th percentiles; *p*-values were calculated by unpaired *t*-test. Micronodules (+): presence of micronodules, micronodules (−): absence of micronodules.

In order to explore whether activation of the type I IFN pathway occurs in the context of ATD, type I IFN serum activity was determined by a reporter cell assay (please see Patients and Methods for details) in 39 ATD patients (26 with HT, 12 with GD) and 39 HC of similar age, sex, and race distribution (Table 1). Patients with ATD had increased type I IFN activity compared to HC (mean ± SD: 1.2 ± 0.4 vs. 0.9 ± 0.4, *p* = 0.002) (Figure 1A). Following these findings patients with ATD were subdivided further according to type I IFN activity into those with “high” (13 patients) and “low” (26 patients) IFN score.

**Table 1:** **Demographic characteristics of the study subjects**.

	ATD patients (*n* = 39)	Healthy controls (*n* = 39)	*p*-Value
No. of subjects	39	39	
Mean age ± SD (years)	48.9 ± 14.4	47.6 ± 11.2	ns
Female to male ratio	4.6:1	4.6:1	ns
Mean disease duration ± SD (years)	5 ± 5.2	NA	NA
% Caucasians	100	100	1
No of patients with GD	12	NA	NA
No of patients with Hashitoxicosis	1	NA	NA
No of patients with HT	26	NA	NA

*GD, Grave’s disease; HT, Hashimoto’s disease; ATD, autoimmune thyroid disease; SD, Standard Deviation; NA, not applicable; ns, no significant*.

#### Clinical and serological correlates of type I IFN activity among ATD patients

In order to determine whether high serum type I IFN activity is associated with the presence of any clinical and serological parameters, we compared the “high” and “low”-IFN score ATD patients using bivariate analysis. Comparisons between the two groups were performed for demographic variables, systemic manifestations, thyroid function, and antibodies. As shown in Table 2, anti-Tg antibodies were present in almost all patients of the “high” IFN group, compared to less than 50% of the “low” IFN patients (*p* = 0.013).

**Table 2:** **Comparison of ATD patients with low or high type I IFN activity in bivariate analysis**.

Variable	Type I IFN activity		
	Low (*n* = 26)	High (*n* = 13)	*p*
Mean type I IFN activity score	0.98 ± 0.19	1.6 ± 0.23	<0.0001
**DEMOGRAPHICS**			
Age, years	47.08 ± 15.16	52.91 ± 12.33	0.278
Disease duration, years	4.32 ± 4.78	6.54 ± 6.03	0.318
No of females	22/26 (84.6%)	10/13 (76.9%)	0.666
**SYSTEMIC MANIFESTATIONS**			
Skin manifestations	8/25 (32%)	7/13 (53.8%)	0.295
Musculoskeletal manifestations	7/25 (28%)	6/13 (46.1%)	0.30
Oral ulcers	6/25 (24%)	0/11 (0%)	0.147
Raynaud’s phenomenon	4/25 (16%)	1/13 (7%)	0.642
Sicca symptoms	5/25 (20%)	3/13 (23.1%)	1
Cardiopulmonary	2/25 (8%)	6/13 (46.1%)	0.011
Renal	0/25 (0%)	0/12 (0%)	1
Hematological	0/24 (0%)	0/11 (0%)	1
Neurologic
Headaches	13/25 (52%)	2/13 (15.3%)	0.039
Other*	2/25 (8%)	1/13 (7.7%)	1
**ABS AGAINST THYROID ANTIGENS**			
Positive anti-Tg autoAbs	12/25 (48%)	11/12 (91.6%)	0.013
Positive anti-TPO autoAbs	21/25 (84%)	11/12 (91.6%)	1
**TYPE OF THYROID DISORDER**			
Graves’ disease	8/26 (30.7%)	4/13 (30.7%)	1
Hashimoto’s/Hashitoxicosis	18/26 (69.3%)	9/13 (69.3%)	1
**THYROID FUNCTION TESTS**			
High TSH	12/26 (46.1%)	8/13 (61.5%)	0.5
Low TSH	9/26 (34.6.9%)	4/13 (30.7%)	1

Other* (includes stroke, white matter microangiopathy, transverse myelitis, cranial/peripheral neuropathy).

Cardiopulmonary manifestations were significantly more frequent in the “high”-IFN ATD group (46 vs. 8%, *p* = 0.011) and included shortness of breath on exertion in two individuals (possibly related to heart failure), pericarditis in one individual, asthma in two cases, and interstitial lung disease in another one. In the low IFN group two cases of asthma were reported. Autoantibodies against Ro/SSA and RNP/Sm nucleoproteins, previously shown to be associated with high type I IFN activity in SLE patients (22), were negative in all ATD subjects (data not shown). No correlations were found between type I IFN levels and autoantibodies to TSHR (*r* = −0.163, *p* = 0.396) by Spearman’s correlation test. Logistic regression analysis revealed an independent association of high IFN-α activity with the presence of anti-Tg antibodies (OR = 17.69, 95% CI: 2.05–560.9) and cardiopulmonary manifestations (OR = 15.34, 95% CI: 1.95–335.5) respectively.

#### Type I IFN activity and thyroid ultrasonographic pattern

We next sought to explore whether type I IFN activity serum levels were associated with ultrasonographic parameters of the thyroid gland. While no significant associations were detected between type I IFN levels and the various ultrasonographic indices examined including thyroid volume, vascularity and hypogenicity indexes, macronodules and inferior thyroid artery blood flow (data not shown), higher type I IFN activity was found in patients with ATD and thyroid micronodules (*p* = 0.04) (Figure 1B).

#### IFIH1 genotypes and IFN levels in ATD patients

To determine whether the IFIH1 risk variant is associated with higher type I IFN levels, the rs1990760 SNP in IFIH1 was genotyped. No association was found between genotypes and type I IFN levels (data not shown). However, a trend toward higher prevalence of the T risk allele among patients with low TSH levels compared to those with high TSH levels (77.7 vs. 50%, OR = 3.5, CI: 0.9–13.3, *p* = 0.07) was found, consistent with the previously reported association of the T allele of the IFIH1 with GD (10).

### T1DM cohort

#### Type I IFN activity in T1DM patients and controls-clinical and laboratory correlates

**Figure 2:**
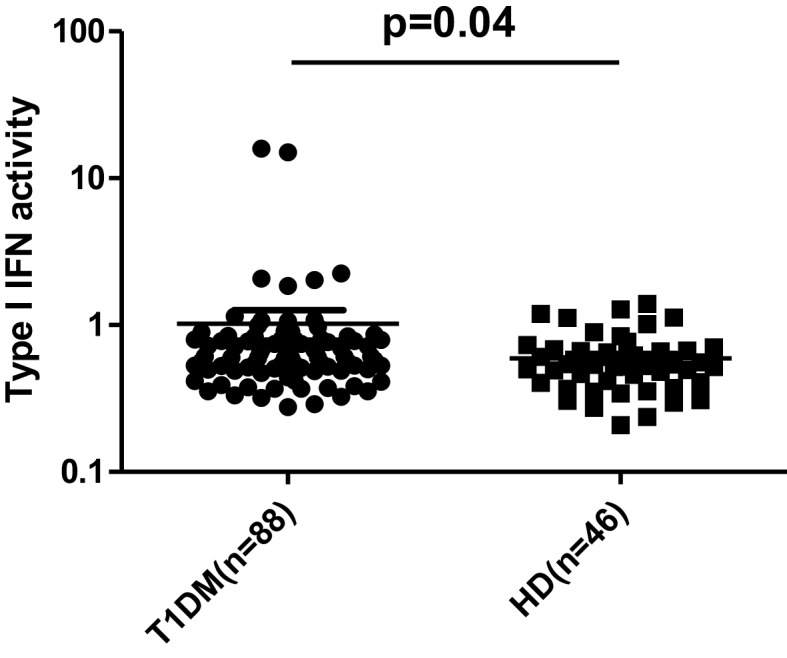
**Increased type I IFN activity in patients with type 1 diabetes mellitus (T1DM) (*n* = 88) compared to healthy donors (HD) (*n* = 46)**. Serum type I IFN activity was assessed using a sensitive reporter cell assay as previously described. Symbols represent individual subjects; horizontal lines represent the mean; *p*-values were calculated by non-parametric Mann–Whitney test.

In order to investigate whether type I IFN pathway is activated in the setting of T1DM, serum type I IFN activity was determined in 88 pediatric T1DM patients and 46 controls of similar age, sex, and body mass index (BMI) distribution (Table 3) by the previously described reporter cell assay. As shown in Figure 2, type I IFN activity was found to be significantly increased in pediatric patients with T1DM compared to age-sex matched controls (mean ± SD:1.1 ± 2.2 vs. 0.6 ± 0.3, *p* = 0.04). Of interest, apolipoprotein-B levels were higher in T1DM patients with high type I IFN activity. No other associations with clinical and/or serological data were observed (Table 4).

**Table 3:** **Characteristics of the T1DM patients and healthy subjects**.

	Healthy subjects (*n* = 46)	T1DM patients (*n* = 88)	*p*
Age (years)	10.53 ± 0.64	12.12 ± 0.57	ns
Female to male ratio	0.8:1	1.1:1	ns
Body mass index (kg/m^2^)	21.16 ± 0.20	20.79 ± 0.58	ns
Diabetes duration (months)	–	57.82 ± 7.83 (0–184)	NA

*ns, no significant; NA, not applicable*.

**Table 4:** **Comparison of TIDM patients with low or high type I IFN activity in bivariate analysis**.

Variable	Type I IFN activity		
	Low (*n* = 72)	High (*n* = 16)	*p*
Mean type I IFN activity score	0.57 ± 0.15	3.06 ± 4.86	<0.0001
Age (years)	11.96 ± 0.74	13.00 ± 1.03	0.500
Female to male ratio	1.2	1.3	1
Body mass index (kg/m^2^)	20.90 ± 0.67	20.21 ± 1.43	0.640
Diabetes duration (months)	59.19 ± 9.25	48.45 ± 17.26	0.583
Total cholesterol (mg/dL)	165.51 ± 5.31	167.27 ± 7.61	0.869
HDL (mg/dL)	59.16 ± 1.74	59.55 ± 2.94	0.915
LDL (mg/dL)	91.95 ± 4.06	90.00 ± 5.48	0.810
Total triglycerides (mg/dL)	70.51 ± 6.31	73.00 ± 10.03	0.847
Lipoprotein (a) (mg/dL)	12.86 ± 1.84	13.50 ± 3.62	0.873
Apolipoprotein-A (mg/dL)	126.46 ± 4.85	137.57 ± 10.70	0.305
Apolipoprotein-B (mg/dL)	81.63 ± 4.16	91.43 ± 1.62	0.037
Urine microalbumin (mg/L)	7.33 ± 0.99	6.83 ± 1.73	0.813
Serum creatinine (mg/dL)	0.64 ± 0.021	0.65 ± 0.06	0.977
Serum urea (mg/dL)	29.59 ± 1.08	31.73 ± 4.94	0.681
CRP (mg/dL)	5.00 ± 1.21	5.91 ± 3.03	0.744
HbA1c (%)	8.08 ± 0.27	7.77 ± 0.42	0.599

## Discussion

While several lines of evidence suggest a central role for the type I IFN pathway in the pathogenesis of a number of systemic autoimmune disorders, more limited data are available regarding its contribution to the pathogenesis of organ-specific autoimmune disease and its potential association with distinct clinical or serological phenotypes. The current study provides evidence of elevated serum type I IFN activity in approximately one third of patients with ATD and one fifth of those with T1DM, using a functional IFN assay.

Designation of ATD and T1DM patients as patients with “high” or “low” type I IFN activity allowed us to test the hypothesis that type I IFN pathway activation identifies ATD and T1DM patients with distinct clinical and serological characteristics. Following both bivariate and multivariate analysis, the presence of anti-TG, but not anti-TPO was associated with a high IFN status among ATD patients. Of interest, a recent study identified SNP (_1623A/G) of the TG gene – previously identified as a major ATD susceptible variant – to modify a binding site for the IFN-induced transcription factor interferon regulatory factor-1 (IRF-1), leading to increased promoter activity and increased TG levels, a major antigenic target for ATD (23). However, the presence of such genetic variant was not assessed in our patient population. Additionally, stimulation of rat thyroidal cells with IFN-α has been shown to lead to persistent (up to 48 h) increase of TG levels through TG promoter activation suggesting that IFN-induced upregulation of the TG autoantigen could lead to generation of antigen specific serum reactivities. Though upregulation of TPO was also observed, this was limited to 24 h and seemed to be independent from the activation of the TPO promoter (24). As no antibodies against Ro/SSA and RNP/Sm antigens were found, those antibodies cannot be implicated in the induction of type I IFN, as has been proposed for SLE (22).

Interestingly, high IFN status was also associated with increased prevalence of cardiopulmonary features. Although IFN-α has been previously linked to atherosclerotic risk and a negative effect of IFN-α on vascular endothelial cells has been demonstrated (25, 26), the pathophysiological implication of this association in ATD will require further investigation. ATD patients with increased type I IFN levels exhibited mostly micronodular sonographic appearance which is related to the presence of lymphocytic aggregates with germinal centers and/or transformed follicular oxyphilic cells (27). Given that type I IFN has been previously associated with B-cell activation and immunoglobulin class switching (28), the relation of micronodulation to type I IFN activity could reflect the contribution of the latter in the pathophysiology of ATD disease. Whilst no significant associations were detected between levels of type I IFN activity and genotypes of the IFIH1 gene, a trend toward increased prevalence of the T risk allele among patients with low TSH levels (defined as<0.5 IU/mL) compared to those with high TSH levels was noted. This finding is consistent with the previously reported association of the T risk allele of the IFIH1 with GD (10).

The association between type I IFN and thyroid disease was first appreciated in 1985 in patients treated with IFN-α for breast cancer (29). Since then, a large number of studies have revealed a high incidence of thyroid abnormalities in IFN-α treated patients, ranging from development of thyroid autoantibodies to overt ATD such as GD or HD (30). IFN-α treatment has been shown to exacerbate preexisting thyroid autoimmunity by increasing the titers of antithyroid antibodies (31). Although unclear, the potential mechanisms through which IFN-α might promote the development of thyroid autoimmunity are multiple, including facilitation of antigen presentation through increased expression of the adhesion and costimulatory molecules ICAM-1, B7.1, and MHC class I antigens on thyrocytes, activation of cytotoxic T-cells, promotion of autoantibody production through direct and indirect effects on B-cell and immunoglobulin class switching, upregulation of thyroid specific antigens, and direct toxicity on thyroidal cells (18, 24). Recently, functional sensors detecting both exogenous and endogenous signals were detected in thyroid cells promoting induction of innate immune responses including activation of type I IFN pathway. In particular, stimulation of thyroid cells with Toll-like receptor ligands led to activation of the interferon-beta (IFN-β) promoter (32). Though therapeutic administration of IFN-α in patients with HCV infection has been mainly associated with induction of ATD, several reports derived from the multiple sclerosis (MS) literature suggest IFN-β as an inducer of ATD among MS patients possibly through stimulation of CXCL10 secretion by thyrocytes (33, 34). Unfortunately, in the present study, exploring whether type I IFN activity was mainly related to IFN-α or IFN-β components was not included in the initial design of the study.

Recent findings have revealed an upregulation of IFN-α inducible genes in peripheral blood mononuclear cells in patients with GD, which correlated with TSHR messenger RNA and protein levels of HLA-DR and IFN-α (35). Although stimulation of primary cultured thyrocytes with recombinant human IFN-α resulted in increased expression of MHC-II antigens and TSHR in these patients, no serum IFN type I activity was detected in the samples tested. IFN-α levels – measured by a commercially available enzyme immunoassay – were also found to be increased in a small cohort of patients with several thyroid disorders including 12 Grave’s disease and four patients with HT. No associations with distinct clinical, serological, or imaging findings were reported (36). Finally, data from a recent report revealed heightened levels of the type I IFN-inducible myxovirus resistance protein A in thyroid tissue derived from early HT patients further reinforcing the implication of the type I IFN pathway in ATD pathogenesis (37).

Activation of the type I IFN pathway was also confirmed in our pediatric diabetic cohort – the largest so far tested, with approximately 20% of patients demonstrating raised serum type I IFN levels, by a sensitive bioassay. The contribution of type I IFN in the pathogenesis of autoimmune T1DM has been previously postulated in both human and animal studies (38–40). Earlier data from a relatively small cohort of mixed adult and pediatric populations demonstrated raised IFN-α in peripheral blood at the mRNA and protein level which correlated well with blood enteroviral RNA, implying a role of enteroviral infection in the pathogenesis of T1DM (39). No clinical or serological associations of type I IFN pathway with disease related biomarkers were reported in that study. On the other hand, endogenous nucleic acids derived from apoptotic pancreatic β cells were also proposed as potential triggers of IFN-α production by plasmacytoid dendritic cells, leading to activation of autoreactive CD4+ T-cells which ultimately lead to destruction of insulin producing pancreatic islets (41). Of interest, in a recently reported animal model of virus induced T1DM, defective function of viral sensors with impaired type I responses was associated with development of the disease and was related to defective clearance of a virus directed against the beta cells of the pancreas (42).

In the current study, apolipoprotein-B levels – previously shown to be associated with pronounced atherosclerotic risk (43, 44) – were increased in the high IFN group in our T1DM cohort. Of interest, apolipoprotein-B is a member of the APOBEC family of proteins, many of which are regulated by type I interferons and particularly IFN-α (45, 46). Whilst it is not known whether increased apolipoprotein-B levels are directly induced by type I IFN, such a probability remains, providing an additional mechanism by which type I IFN might contribute to the pathogenesis of atherosclerosis (47).

The demonstration of type I IFN activity in serum of patients with ATD and T1DM, supports its role in the pathogenesis of both organ-specific and systemic autoimmune disorders. While the reasons for the tissue specificity in the autoimmune process remain elusive, the identification of the type I IFN pathway as a common pathogenetic denominator among distinct and diverse autoimmune phenotypes may explain the similarities found in patients with IFN-related disorders, such as strong familial aggregation and female predominance, and contribute to the identification of unifying underlying determinants of autoimmunity. Familial aggregation of both high IFN and ATD in SLE families could support a case for IFN being causal in both (48).

In conclusion, the findings of the present study support a role for type I IFN in the pathogenesis of organ-specific autoimmune disorders, particularly in ATD and T1DM patients. These data extend our current list of type I IFN-related autoimmune disorders and may provide insight into shared pathogenic factors and suggest new targets for therapeutic intervention.

## Conflict of Interest Statement

The authors declare that the research was conducted in the absence of any commercial or financial relationships that could be construed as a potential conflict of interest.
